# Mapping autism in Egypt: population-based insights into prevalence, risk determinants, and severity among children aged 1–12 years

**DOI:** 10.1186/s13229-025-00665-1

**Published:** 2025-05-29

**Authors:** Ammal M. Metwally, Ebtissam M. Salah El-Din, Samia M. Sami, Ehab R. Abdelraouf, Sara F. Sallam, Amal Elsaeid, Mostafa M. El-Saied, Engy A. Ashaat, Asmaa M. Fathy, Hazem M. El-Hariri, Ghada A. Elshaarawy, Maysa S. Nassar, Manal A. Shehata, Inas R. El-Alameey, Randa I. Bassiouni, Mohamed H. Abdou, Mona A. Helmy, Nahed A. Elghareeb, Mohamed AbdAllah, Thanaa M. Rabah, Somia I. Salama, Rehan M. Saleh, Lobna A. El Etreby, Dalia M. Elmosalami, Eman Eltahlawy, Dina Abu Zeid

**Affiliations:** 1 Community Medicine Research Department, Medical Research and Clinical Studies Institute, National Research Centre (Affiliation ID:60014618), P.O. 12622, Dokki, Cairo, Egypt; 2 Child Health Department, Medical Research and Clinical Studies Institute, National Research Centre (Affiliation ID: 60014618), Dokki, Cairo, Egypt; 3 Child With Special Needs Department, Medical Research and Clinical Studies Institute, National Research Centre (Affiliation ID: 60014618), Dokki, Cairo, Egypt; 4 Clinical Genetics Department, Human Genetics and Genome Research Institute, National Research Centre (Affiliation ID: 60014618), Dokki, Cairo, Egypt; 5 Mansoura Health Directorate, Ministry of Health and Population, Dakhlyia, Egypt; 6 Environmental and Occupational Medicine Department, Environmental and Climate Change Research Institute, National Research Centre (Affiliation ID: 60014618), Dokki, Cairo, Egypt; 7 Prevention of Disability General Directorate, Ministry of Health and Population, Cairo, Egypt; 8 Complementary Medicine Department, Medical Research and Clinical Studies Institute, National Research Centre (Affiliation ID: 60014618), Dokki, Cairo, Egypt

**Keywords:** Autism spectrum disorder, Prevalence, National survey, Risk factors, Severity

## Abstract

**Background:**

The prevalence of autism spectrum disorder (ASD), a common developmental disorder, has surged in recent years. Accordingly, the identification and early management of possible risk factors can diminish ASD incidence.

**Aim:**

To determine the prevalence and severity of idiopathic ASD in Egyptian children aged 12 months to 12 years, and to identify the epidemiological, sociodemographic, and environmental risk factors contributing to this disorder.

**Methods:**

This study comprised 41,640 children from the main eight geographic areas in Egypt. It was conducted through four phases: household screening, facility-based screening for high-risk children, diagnosis confirmation, and risk factor assessment.

**Results:**

The prevalence of ASD as confirmed by the criteria of the Diagnostic and Statistical Manual of Mental Disorders, Fifth Edition (DSM-5) and the Childhood Autism Rating Scale (CARS) was 1.1% (455 out of 41,640), with significant geographic variability. Urban areas had a significantly higher prevalence than rural areas. Children aged 3–6 years showed the highest prevalence at 1.5%. Boys were four times more affected than girls, with prevalence rates of 1.7% and 0.4%, respectively. Significant risk factors included: a history of convulsions (AOR = 4.7; 95% CI: 3.3–6.79), low birth weight (AOR = 2.08; 95% CI: 1.54–2.79), *prolonged* stays in neonatal intensive care unit (NICU) longer than two days (AOR = 1.91; 95% CI: 1.46–2.49) and maternal health problems during pregnancy (AOR = 1.66; 95% CI:1.36–1.95). Regarding severity, 45% of diagnosed children had moderate ASD, 39% had severe ASD, and 16% had mild ASD. Female gender and older age were significant predictors of greater ASD severity.

**Conclusion:**

ASD prevalence in Egypt is comparable to other Middle Eastern countries. Policymakers should utilize these findings to design targeted public health interventions aimed at early detection, management, and prevention of ASD progression.

**Supplementary Information:**

The online version contains supplementary material available at 10.1186/s13229-025-00665-1.

## Introduction

Autism spectrum disorder (ASD) is a lifelong neurodevelopmental disorder characterized by persistent difficulties in initiating and maintaining reciprocal social interactions and communication [1, 2]. It is also marked by restricted, repetitive, and inflexible behaviors, interests, or activities that are disproportionate to the individual’s age and sociocultural context [1, 3]. Diagnosis typically occurs in childhood, with noticeable signs as early as 18 months of age [4].

ASD imposes significant physical, psychological, and emotional challenges on affected children and their families [5]. Children with ASD often experience high levels of stress, sleep disturbances, executive function difficulties, and an increased risk of mental health issues [6]. Studies have also reported high levels of parental stress, depression, anxiety, and physical health complications [7, 8]. Furthermore, ASD presents a substantial economic burden on both families and national healthcare systems [9–13]. Therefore, early detection and intervention are crucial for improving the intellectual and behavioral outcomes of affected children while reducing the burden on families [14].

Epidemiological studies worldwide indicate an increasing trend in ASD prevalence. This rise reflects not only a genuine increase in cases but also heightened awareness, improved research methodologies, and evolving diagnostic criteria [3]. In 2020, ASD prevalence in the United States was estimated at 1 in 36 children [15]. Similarly, prevalence rates range from 3.9% in Asia [16] to 0.38–1.55% in Europe [17] and 0.6% in Southeast Asia [18], with notable increases observed in countries like China [19]. In the Middle East, although data is limited, reported prevalence ranges from 0.11% in Iran to 1.53% in Lebanon [20].

Despite rising global prevalence, community-based research on ASD prevalence and its risk factors in Egypt remains limited. Previous estimates suggested a prevalence of 3.3% for children at high risk of autism [21], aligning with facility-based studies that reported rates of 3.4% [22] and 2.8% [23]. These findings highlight the need for comprehensive, population-based studies to better understand ASD prevalence and associated risk factors within the Egyptian context.

Several factors contribute to an increased risk of ASD, including genetic, epigenetic, and environmental influences [24, 25]. In fact, many known risk factors can be prevented or modified [26]. Some proposed risk factors require further research, such as parental age at conception, prenatal factors (including maternal bleeding and medication use during pregnancy), gestational diabetes, and hypertension. Additionally, neonatal factors such as prematurity and fetal hypoxia have also been implicated [27, 28].

The primary aim of this study was to determine the national prevalence of non-syndromic or idiopathic ASD and its severity levels among Egyptian children aged 1–12 years through a community and facility-based survey. Secondary objectives included estimating ASD prevalence in eight governorates and identifying epidemiological, sociodemographic, and medical risk factors associated with ASD in Egypt. The findings from this study can contribute to global discussions on the rising prevalence of ASD by adding a crucial data point from one of the Middle Eastern countries (Egypt) where autism research is still in its infancy. By identifying significant risk factors, this study will reinforce the need for early intervention programs that are tailored to local contexts. Furthermore, it will enable policymakers in Egypt to better align with global best practices in ASD management and prevention, addressing both the specific needs of the Egyptian population and the broader implications for public health strategies in the region.

## Materials and methods

### Study type, design and setting

This community- and facility-based study was part of a national survey in Egypt, aimed at estimating the prevalence of autism spectrum disorder (ASD) among children aged 1–12 years. The survey was conducted in four phases. Phase one consisted of a household-based survey designed to identify children with developmental deviations who were potentially at risk for ASD. In phase two, these children were referred to maternal and child health centers, where they were screened for ASD using appropriate screening tools. Phase three involved the formal diagnosis of ASD based on the DSM-5 criteria and the Childhood Autism Rating Scale (CARS). In the final phase, children with confirmed ASD diagnoses were assessed for intellectual functions and risk factors, including sociodemographic, environmental, prenatal, and neonatal factors.

The study was conducted in eight governorates, representing Egypt’s major geographic regions based on population density. It spanned four years, from December 2017 to December 2021.

### Target group

#### Justification for the selected age range

There isn’t a particular age at which autism peaks for every child. The time of emergence of early signs of ASD can vary widely from child to child. Some children show early signs of autism from 12 months onwards, for example, when the cortical subplate (a transient brain structure) has largely disappeared in the cortical association areas and the cerebellar external granular layer has vanished [29]. Others may not exhibit signs of autism until they are 24 months old or older. Currently the average age at the diagnosis of ASD has been reported at 43 months [30]. The American Academy of Pediatrics recommends that all children have a particular screening for ASD at the ages of 18 and 24 months, as part of routine well-child visits [4]. It is possible to identify ASD in children as young as 18 months. By the age of two, a diagnosis made by a qualified specialist is considered reliable [31]. However, a lot of children don’t get a definitive diagnosis until they’re much older. Consequently, the age range from 1 to 12 years was deemed appropriate for identifying new and overlooked children with ASD.

#### Detailed rationale for age range selection

The study focused on three age categories; infants and toddlers aged from 1 year to three years for early detection, ages from three to under 6 years denoting established cases. The third age category included the school age 6–12 years for monitoring trend changes.

#### Inclusion and exclusion criteria

Inclusion criteria: Any child in the age range of 1–12 years was included, whether was designated by parents as typically developed or had delayed milestones for his age, as well as any child who met the definition of autism [1] whether previously identified as idiopathic ASD or newly suspected cases during the current survey.

Exclusion criteria: Children with known or previously diagnosed genetic disorders (e.g., Down syndrome, Turner syndrome, or fragile-X syndrome) or with disabilities affecting vision, and hearing, or children with movement disorders due to isolated orthopedic problems, or congenital deformities of limbs, all were excluded from the survey. The validated WHO ten-question screening tool was explained to households to detect disabilities affecting hearing, vision, movement, learning, thinking, or social relationships [32]. Parents were asked to provide genetic test results or official documentation of these disabilities if available. The researchers ensured that children with detected disabilities or developmental delays other than ASD were enrolled in rehabilitation programs by the Ministry of Health.

This manuscript is a part of a large project titled “National Prevalence Survey for Autism Spectrum Disorders (ASDs): Assessing its Epidemiological Pattern and Risk Factors.” Recent publications have documented data derived from this project concerned with the prevalence of developmental delays and developmental disabilities in Egyptian children [33–37].

Children diagnosed with ASD were referred for management to the outpatient clinics for Children with Special Needs and the Clinical Genetics Department at the National Research Centre, Egypt.

### Sampling frame and cluster preparation

The sampling process was conducted in three stages, utilizing three distinct sampling frames. The first frame consisted of the comprehensive list of Egypt’s 27 governorates, as defined by the 2017 census from the Central Agency for Public Mobilization and Statistics (CAPMS) [38]. Population distribution as presented in the 2017 census data [39] showed that 17.1% of the population resided in urban governorates, 43% in Lower Egypt, 38% in Upper Egypt, and only 1.7% in Frontier governorates.

Stage 1: Governorate Selection: In the first stage, a representative sample was drawn from eight governorates, each representing one of Egypt’s major geographic regions. These included: One urban governorate (Cairo), three governorates from Upper Egypt (Fayoum, Assiut, and Aswan), three governorates from Lower Egypt (Damietta, Dakahlia, and Gharbia), One Frontier (border) governorate (Marsa Matrouh). Refer to Supplementary Fig. 1 for a map showing the distribution of Egypt’s 27 governorates across four geographic regions (adapted using data from the Humanitarian Data Exchange under the CC BY-IGO license [40].

Stage 2: City and Village Selection: In the second stage, a representative sample of urban cities (kism) and rural village units (markaz) was selected from each of the chosen governorates. Egypt is subdivided into 177 cities and 162 local village units [41]. To ensure a diverse and representative sample, each governorate was categorized into low, medium, and high according to its human development scores [41]. From each category within each governorate, one city for urban areas and one local unit for rural areas were randomly selected.

Stage 3: Cluster Selection: In the third stage, all shiakhas (urban neighborhoods) and villages from the randomly selected kism and markaz were listed as clusters. One shiakha and one village were randomly chosen from each social category within the selected governorates. This approach resulted in a final sample of 45 blocks, including 24 urban kism and 21 rural markaz, ensuring heterogeneity in the data collected (S-Table 1).

In this stage, households within the selected city and village blocks were identified. The sample size was allocated proportionally based on the population size of large governorates. For governorates with smaller populations, arbitrary sample sizes were used, with weighting adjustments applied during data analysis to account for these discrepancies***.***

### Calculation of sample size

The sample size calculation was based on the previously estimated prevalence of ASD in the North Africa and Middle East (NAME) region. The age standardized prevalence rate was 303.6 (250.4–365.5) and 304.4 (251.2–366.1) per 100,000 in 1990 and 2019, respectively [42]. These prevalence rates were considered to determine the largest sample size necessary, with a margin of error set at 0.0049, a confidence level of 95%, and a minimum reliability of 80% for the least reliable questionnaire used in ASD detection. Based on these calculations, a target of 40,000 children was estimated to ensure data accuracy and provide reliable prevalence estimates for ASD [43]. Ultimately, the actual number of children included in the study was 41,640, derived from 22,026 surveyed households across 45 blocks in the eight targeted governorates, were structured to provide a nationally representative estimate for ASD prevalence, ensuring that the findings could be generalized to the population within the selected age groups. The study reached the following target groups: 8,382 infants and toddlers (1–3 years) for early detection; 12,933 preschool children (> 3–6 years) for confirmation and 20,324 school children (> 6–12 years) for monitoring trend and assessing intervention outcomes. The sample included 21,437 males and 20,202 females, providing a balanced gender distribution for accurate prevalence estimation.

### Phases and instruments of the survey

The current survey study was done as four phases after preparation:

#### Phase I: household screening for children with features suspected of ASD

This phase involved screening children for developmental delays and signs suspicious of ASD at the household level. The Vineland Adaptive Behavior Scales (VABS) [44] were used for diagnosing intellectual and developmental disabilities, including ASD. Adaptive behavior assessment provides important behavioral markers and targets for diagnosis and intervention. Children with ASD show substantial difficulties in adaptive behavior domains, particularly in socialization, even with normal intelligence [45].

The VABS produces an overall composite standard score and age-equivalent standard scores in four domains: communication, daily living skills, socialization, and motor skills. It has been shown to provide reliable assessments, with interrater reliability coefficients of 0.74, test–retest reliability coefficients between 0.81 and 0.86, and split-half reliability coefficients ranging from 0.83 to 0.94 across all domains [46].

The Arabic version of the Vineland Adaptive Behavior Scales (VABSA) was employed [47], which has been validated with strong reliability and used in several studies across Arab countries [22, 48]. The VABS measures adaptive behavior, with a mean composite score of 100 and a standard deviation of 15. Children scoring below 70, indicating developmental delays or potential autism. To prevent overlooking ASD cases, children with below-average scores (< 85) were referred to maternal and child health centers for further evaluation using specialized ASD assessment tools.

### Phase II: facility-based screening for high-risk children (suspected of autism)

- Children identified as at risk for autism in Phase I were referred to maternal and child health care facilities for further screening. The tools used in this phase included: M-Chat for children aged 1–3 years [49, 50] Gilliam Autism Rating scale (GARS-2) [51] for children 3–12 years. Previous studies have reported adequate sensitivity and specificity of the M-CHAT ranging from 87 to 91% [51] and adequate internal reliability for the entire questionnaire of M-CHAT (α = 0.85) [52]. On the other hand, the internal consistency of the four original sub scales of GARS-2 were 0.82 for Stereotyped Behavior, 0.84 for Communication, 0.85 for Social Interaction, and 0.68 for Developmental Disturbance. Test–retest reliability ranged from 70 to 0.90 [53].
- Additionally, the Denver II Developmental Screening Test was used to assess children aged 1 to 6 years. The sensitivity and specificity of the Denver II were reported to be 0.83 and 0.43, respectively [54]. Children identified as high-risk for ASD based on these assessments were subjected to further investigation in Phase III, with early results already published by the research team [23].

#### Phase III: confirmatory diagnostic of Autism

This phase focused on conducting in-depth interviews and evaluations with children identified as high-risk in Phase II. ASD diagnosis was based on the criteria outlined in the DSM-5**,** with severity levels determined by impairments in social communication and the presence of restricted, repetitive behaviors. Autism severity was classified into three levels based on the level of support needed: Level 3, which indicates a requirement for very substantial support; Level 2, requiring substantial support; and Level 1, requiring support. [1].

In addition to the DSM-5 criteria, the Childhood Autism Rating Scale (CARS) was employed to assess autism severity. The CARS is among the most commonly utilized assessment tools for autism. Various studies have evaluated the internal consistency of the CARS by measuring Cronbach’s alpha, yielding values ranging from 0.82 to 0.95 [55]. Park and Kim [56] examined the construct validity of the CARS in the context of DSM-5 criteria and determined that the two-factor model had strong fit indices. It shows solid reliability and validity. The CARS is a rating scale completed by clinicians with four frequency levels from 1 to 4 based on observations of individuals and accessible information. The CARS is a behavioral rating instrument, consisting of 15 items, that is consistently employed to quantitatively assess the severity of suspected ASD symptoms [57]. According to the CARS manual, ASD is defined as a CARS score of ≥ 30 points. CARS scores between 30 and 36.5 indicate mild to moderate autism, while scores between 37 and 60 suggest severe autism [58].

All instruments used in the current study were Arabic versions and culturally adapted standardized versions of the originally English ones. The Arabic version of the Vineland Adaptive Behavior Scales (VABSA) (46), the Arabic and culturally adapted version of M-CHAT [59], the Arabic and culturally appropriate version of GARS-2 [60] and the Arabic version of CARS [61]. These tools have been used in several studies across Arab countries and have been validated with strong reliability [22, 49, 61–64]. Children diagnosed with developmental delays other than ASD were referred to certified rehabilitation centers for speech therapy, behavior modification, and skill development sessions.

#### Phase IV: assessment of risk factors

After confirming the diagnosis of ASD, phase IV involved the assessment of the intellectual functions of children diagnosed with ASD. The presentation and analysis of the data pertaining to this entity have been covered in a separate manuscript that is currently under publication. This phase also included a comprehensive evaluation of risk factors through face-to-face interviews with parents or caregivers. These factors are: Sociodemographic factors: age, sex, birth order, number of siblings, maternal age, residence, and parental education and occupation.

- Environmental factors: access to water, condition of the house (e.g., paint exposure).
- Maternal health: during pregnancy and family history of psychiatric disorders.
- Pre-, peri-, and neonatal risk factors: associated with ASD.

These interviews gathered critical data on the potential environmental and familial factors contributing to autism risk [65–67].

### Implementers of the survey phases (Fig. 1)

The plan of implementation of all phases of the project is summarized in the following flow chart.

**Fig. 1:**
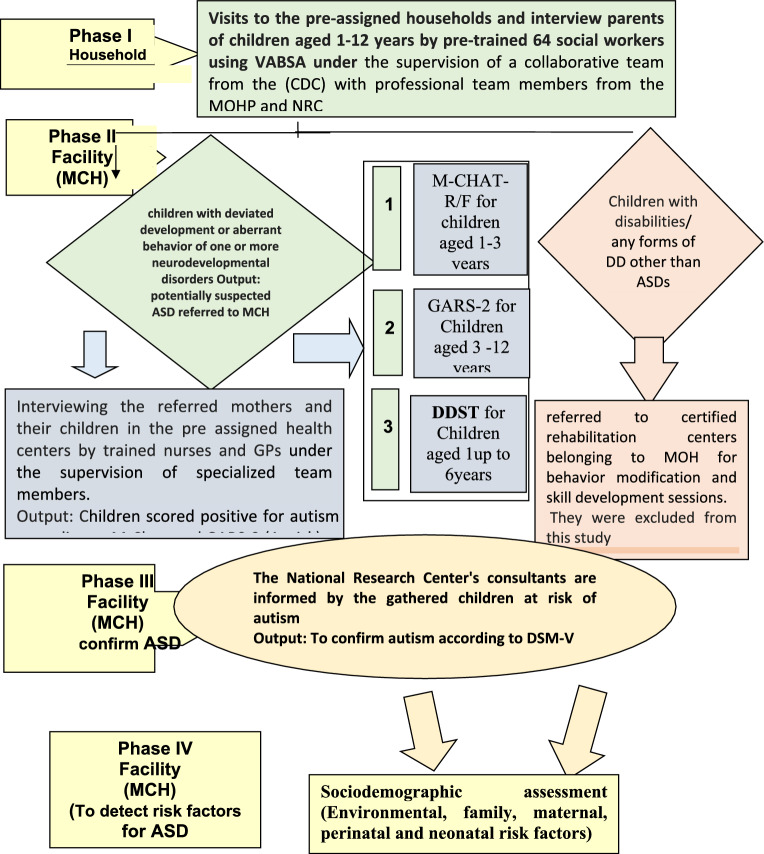
Flow chart of the implementation of the national prevalence survey of ASD

Phase I (Household Screening): The fieldwork for this phase was carried out by pre-trained professional field surveyors from the Cairo Demographic Centre (CDC), selected from staff nominated by the Demographic and Health Survey in Egypt. Sixty-four social workers (6 per governorate) were trained in standardized questionnaire administration. Training of the field surveyors and supervision of the survey was carried out by a collaborative team from the CDC, a specialized psychologist, pediatricians, and public health experts from the National Research Centre (NRC). A pilot study was conducted with 80 participants (10 per governorate) to validate the questionnaire, revise problematic items, and ensure clarity.

Phase II (Facility-Based Screening): This phase was conducted by trained nurses selected from health units and maternal and child healthcare facilities under the Ministry of Health and Population (MOHP). A total of 208 nurses were trained by a specialized psychologist and neurodevelopmental pediatricians from NRC to use autism screening tests and calculate scores. For children who did not visit health facilities, trained nurses arranged outreach visits to ensure reliable testing.

Phase III (Confirmatory Diagnosis): This phase was conducted by specialists from the National Research Centre. Appointments were scheduled to evaluate children suspected of having autism. The evaluation and diagnosis confirmation were carried out by qualified neurodevelopmental -behavioral pediatricians with extensive experience in diagnosing and treating children with ASD. A clinical genetics team focused on confirming or ruling out genetic disorders that presented with distinct physical features or positive laboratory findings.

Phase IV (Risk Factor Assessment): This phase was managed by specialized epidemiologists from the National Research Centre, who conducted detailed interviews with the parents or caregivers of children diagnosed with ASD.

**Fig. 2:**
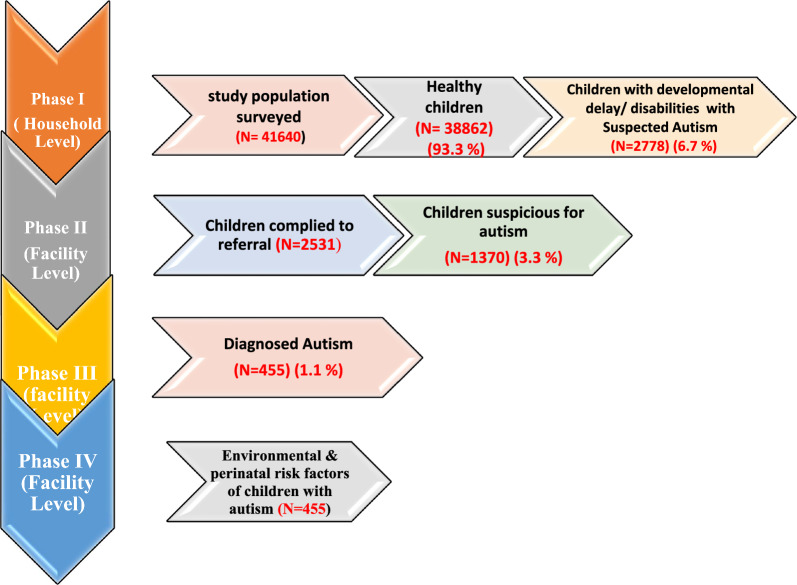
Phases of the study and their output

The four phases of the study and their outputs are summarized in Fig. 2.

Phase I was conducted at the household level, where features of developmental delays or disabilities, including suspected autism, were identified in 6.7% of the surveyed population (41,640 children).

Phases II, III, and IV were carried out at healthcare facilities in consecutive stages.

- The output of Phase II identified children suspected of autism (3.3%) based on the results of the M-CHAT and GARS-2 screening tools.
- Phase III confirmed the diagnosis of autism in these children, utilizing the DSM-V criteria and CARS assessments.
- Phase IV focused on identifying associated risk factors for ASD through a comprehensive evaluation of confirmed cases.

### Statistical methods

Data were entered and analyzed using the Statistical Package for Social Sciences (SPSS), version 25 (IBM Corp., Armonk, NY). Data were presented using mean and standard deviation for quantitative variables and frequency and percentage for qualitative ones. In all calculations, the denominator corresponded to the relevant study population. This was either the total number of children aged 1–12 years enrolled in the study (n = 41,640) or the total number diagnosed with autism (n = 455). For sociodemographic variables related to the parents of the children, the denominator remained the total number of children enrolled in the study. Missing values remained in the denominator but were excluded from the numerator to ensure accurate proportion calculations. This approach was used to ensure that the calculated proportions reflected the actual situation.

Rationale for statistical tests: Given the normal data distribution, statistical tests were selected based on variable type. For two-group comparisons of quantitative variables, an independent t-test was conducted; for comparisons involving more than two groups, ANOVA was used. Qualitative variables were tested using the Chi-square (X^2^) test. Comparisons were assessed using crude odds ratios (COR) with 95% confidence intervals (CI) when comparing ASD and non-ASD children. A p-value of less than 0.05 was considered statistically significant.

Description of Logistic Regression Methods: Logistic regression analysis was performed to assess the contribution of each independent variable in predicting the odds of ASD, using adjusted odds ratios (AOR) [68]. Risk factors were incorporated into the multivariate logistic regression model through the “backward” selection method.

The variables included in the multivariate logistic regression model for predicting ASD were:

1. Sociodemographic factors such as governorate, age, urban/rural locality, maternal and paternal age, parental education level, and other demographic variables.
2. Family history of epilepsy, obsessive–compulsive disorder (OCD), and depression.
3. Maternal risk factors during pregnancy, including infections (e.g., influenza, rubella, CMV), fever, hypertension, diabetes mellitus (DM), smoking, and drug intake during pregnancy (e.g., antihypertensives, hypoglycemic drugs, antiemetics, and antibiotics).
4. Natal and postnatal health issues, such as low oxygen levels during labor, neonatal jaundice, paleness, and the need for cardiopulmonary resuscitation.

The variables included in the logistic regression model predicting the severity of ASD were the same as those for ASD risk factors (1–4), with the addition of: 5. Environmental factors such as the type of house flooring, proximity to industrial areas, garbage dumps, cultivated land, and the storage and use of pesticides. Additional factors included the condition of house painting and the source of drinking water.

The effect sizes for key findings were presented as adjusted odds ratios (AOR) with corresponding confidence intervals (CI).

A significant association was considered when the 95% CI did not include the value of 1.0. Statistical significance was set at a p-value of less than 0.05, with highly significant associations defined as p-values less than 0.01.

Statistical approach for dealing with outliers and potential confounding factors

Outlier detection and management utilized visual, statistical, and influence diagnostics. Boxplots, scatterplots and Z-scores (± 3 SD), identified extreme values in birth weight, parental age, and maternal health factors. Multivariate analyses employed Cook’s Distance and Mahalanobis Distance to detect influential data points, identifying potential outliers that could bias results.

Outliers were first verified for data entry errors. Extreme values deemed biologically plausible were Winsorized (capped at the 1 st and 99 th percentiles) to limit their influence. Outliers were only removed if deemed biologically implausible (e.g., birth weight < 1 kg or > 5 kg in full-term infants).

Sensitivity analyses tested the impact of outlier handling by comparing regression models with and without outliers. Robust regression techniques, including quantile regression, ensured consistency of results. This structured approach minimized bias while preserving statistical validity.

Several sociodemographic, perinatal, and genetic factors acted as confounders in this study, influencing the relationship between ASD severity and risk determinants. To ensure the accuracy of the findings and reduce bias, the study employed several statistical and methodological strategies to control for confounders that could influence the relationship between risk factors and ASD severity. By applying multivariate regression models, stratified analyses, inclusion of key covariates, and sensitivity testing, the study effectively controlled for confounders and strengthened the reliability of its findings.

These statistical methods controlled for confounders, strengthening the study’s credibility on ASD prevalence, risk determinants, and severity.

## Results

Table 1 shows ASD prevalence by sociodemographic characteristics. ASD prevalence was significantly higher in urban (1.57%) than rural areas (0.68%). Children from middle (1.19%) and high (1.1%) socioeconomic classes had the highest prevalence. ASD prevalence was 1.7% in boys—four times higher than in girls (0.4%).

**Table 1:** Prevalence of ASD according to the sociodemographic characteristics of the study population

Socio-demographic parameters	S Surveyed children (n = 41,640) N (%)^¤^	Diagnosed Autism (n = 455; 1.09%)§ N (%)^¤ ¤^	% Percentage within each category
Locality (Urban/Rural)**			
Urban	19,422 (46.6%)	305 (67%)	1.57%
Rural	22,218 (53.4%)	150 (33%)	0.68%
Social class			
Low	13,586 (32.6%)	134 (29.5%)	0.99
Middle	13,887 (33.4%)	165 (36.3%)	1.19
High	14,167 (34.0%)	156 (34.3%)	1.1
*Geographical Distribution by geographical area*			
Cities**	6919 (16.6%)	173 (38.0%)	2.5
Lower Egypt	15,892 (38.2%)	97 (21.3%)	0.61
Upper Egypt	14,344 (34.5%)	144 (31.6%)	1.00
Frontier	4485 (10.8%)	41(9.0%)	0.91
Sex**			
Surveyed boys	21,437 (51.5%)	368 (80.9%)	1.7
Surveyed girls	20,203 (48.5%)	87 (19.1%)	0.4
Age**			
1- < 3 years	8383 (20.1%)	45 (9.9%)	0.54
3- < 6 years	12,933 (31.1%)	194 (42.6%)	1.50
6–12 years	20,324 (48.8%)	216 (47.5%)	1.1
Mean age of Children (1–12 years) ± SD**	6.07 ± 3.11	6.6 ± 2.51	
*Mothers’ age at giving birth (n* = *39,365)#*			
18 years to < 35 years	35,958 (91.3%)	363 (79.8%)	1.01
≥ 35 years	3407 (8.7%)	85 (18.7%)	2.49
*Mothers’ Education*			
Illiterate/below high school	16,046 (38.5%)	274 (60.2%)	1.7
High School	18,609 (44.7%)	161 (35.4%)	0.87
University or higher	6674 (16.0%)	20 (4.4%)	0.3
*Fathers’ Education*			
Illiterate/below high school	14,666 (35.2%)	277 (60.9%)	1.9
High School	18,390 (44.2%)	164 (36.0%)	1.09
University or higher	6662 (16.0%)	14 (3.1%)	0.2
*Mothers’ work*			
Employed	6014 (14.4%)	40 (8.2%)	0.7
Unemployed	35,31 (84.8%)	415 (91.2%)	1.2

^§^ = X^2^ between autism cases and healthy cases, * = sig (< 0.05), ** = highly sig (< 0.01), out of total surveyed children, out of total children with autism, #no mothers in some houses

ASD was most prevalent in children aged 3 to < 6 years (1.5%), followed by those aged 6–12 years (1.1%). These rates were significantly higher than the 0.54% prevalence in children aged 1 to < 3 years.

ASD prevalence was higher in children born to mothers aged ≥ 35 years (2.5%) than in those born to mothers aged 18 to < 35 years (1.0%).

ASD prevalence was highest in children of illiterate parents (mothers: 1.7%, fathers: 1.9%) compared to those whose parents had high school or university education.

Lastly, ASD prevalence did not differ significantly between children of employed (0.7%) and non-employed (1.2%) mothers.

**Fig. 3:**
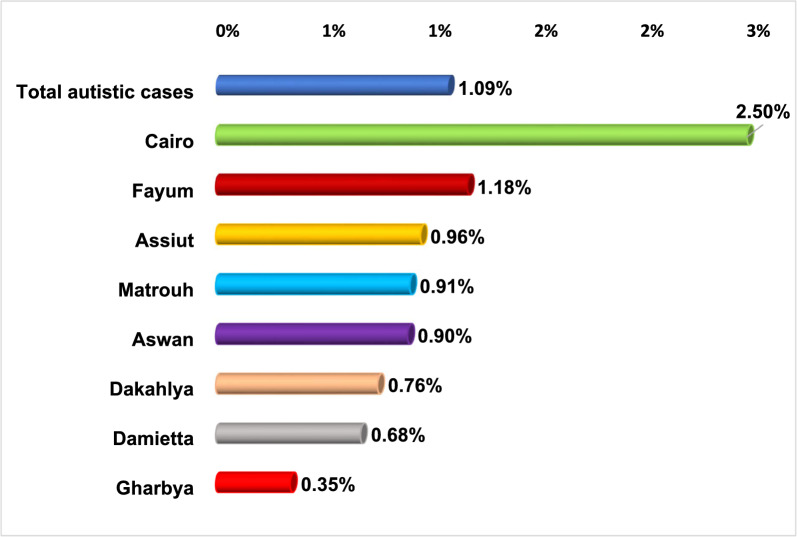
Prevalence of ASD according to the geographical distribution by governorates out of the 41,640 surveyed Egyptian children aged 1–12 years

Figure 3 illustrates autism prevalence by geographical distribution among the 41,640 surveyed Egyptian children aged 1–12 years. Autism prevalence was highest in urban areas, particularly in Cairo (2.5%), followed by Fayoum (1.18%) and Assiut (0.96%). Overall, prevalence was significantly higher in Upper Egypt compared to Lower Egypt.

Table 2 shows that the odds of autism were significantly increased in association with perinatal health problems, whether neonatal or maternal. Neonatal and postnatal factors had the highest odds for autism occurrence. Children with a history of cyanosis after birth had the greatest risk (COR = 25.59, 95% CI: 19.9–32.9), followed by those with low birth weight (COR = 18.1, 95% CI: 14.9–21.9). Additionally, children who experienced convulsions after birth had 12 times the odds of autism (COR = 12.05, 95% CI: 9.0–16.2), and those born prematurely (< 37 weeks gestation) had 10 times the odds (COR = 10.5, 95% CI: 7.5–14.8). Maternal health complications during pregnancy or difficult labor also contributed to the increased risk of ASD, as shown in Table 2.

**Table 2:** Odds of having ASD among children aged 1–12 years according to perinatal health problems

Prenatal, natal and neonatal problems	Surveyed children (n = 41,640) N (%)	Diagnosed Autism (n = 455; 1.09%) § N (%)	COR (CI) autism vs non-autism
Children with history of cyanosis# after birth§§	546 (1.3%)	42(9.2%)	25.59 (19.9–32.9) **
History of low birth weight (LBW) (< 2500 g) §§	1848 (4.4%)	196(43.1%)	18.1 (14.9–21.9) **
Children with history of any convulsions after birth§§	675 (1.6%)	55(30.9%)	12.05 (9.0–16.2) **
History of premature delivery (< 37 weeks gestation) §§	413 (1%)	40 (8.8%)	10.5 (7.5–14.8) **
Children with history of meningitis after birth	378 (0.9%)	29 (6.4%)	9.39 (6.3–13.9) **
Mothers with history of any health problem during pregnancy	2770 (6.6%)	75 (16.5%)	2.8 (2.19–3.6) *
Children kept in an incubator for more than two days§	3078 (7.4%)	126 (27.7%)	1.97 (1.5–2.61) *
Difficult labor	6092 (14.6%)	92 (20.2%)	1.52 (1.2–1.92) *
Children with history of jaundice after birth§	11,028 (26.5%)	141 (31%)	1.3 (1.1–1.59) *

^§^ = X^2^ between children with autism and healthy children, * = sig (< 0.05), ** = highly sig (< 0.01),

^§^Mothers having complications during pregnancy such as gestational diabetes, hypertension, iron deficiency anemia, anxiety, depression, or infection[50]

^§§^Difficult labor refers to prolongation in the duration of labor, especially in the first stage of labor. It can be a contributor to maternal mortality and morbidity if unrecognized or untreated [51]

^#^Cyanosis is a bluish discoloration of the skin, lips, or nails caused by low oxygen levels in the blood [69]. It occurs when deoxygenated hemoglobin in the blood exceeds 5 g/dL, leading to reduced oxygen delivery to tissues

**Fig. 4:**
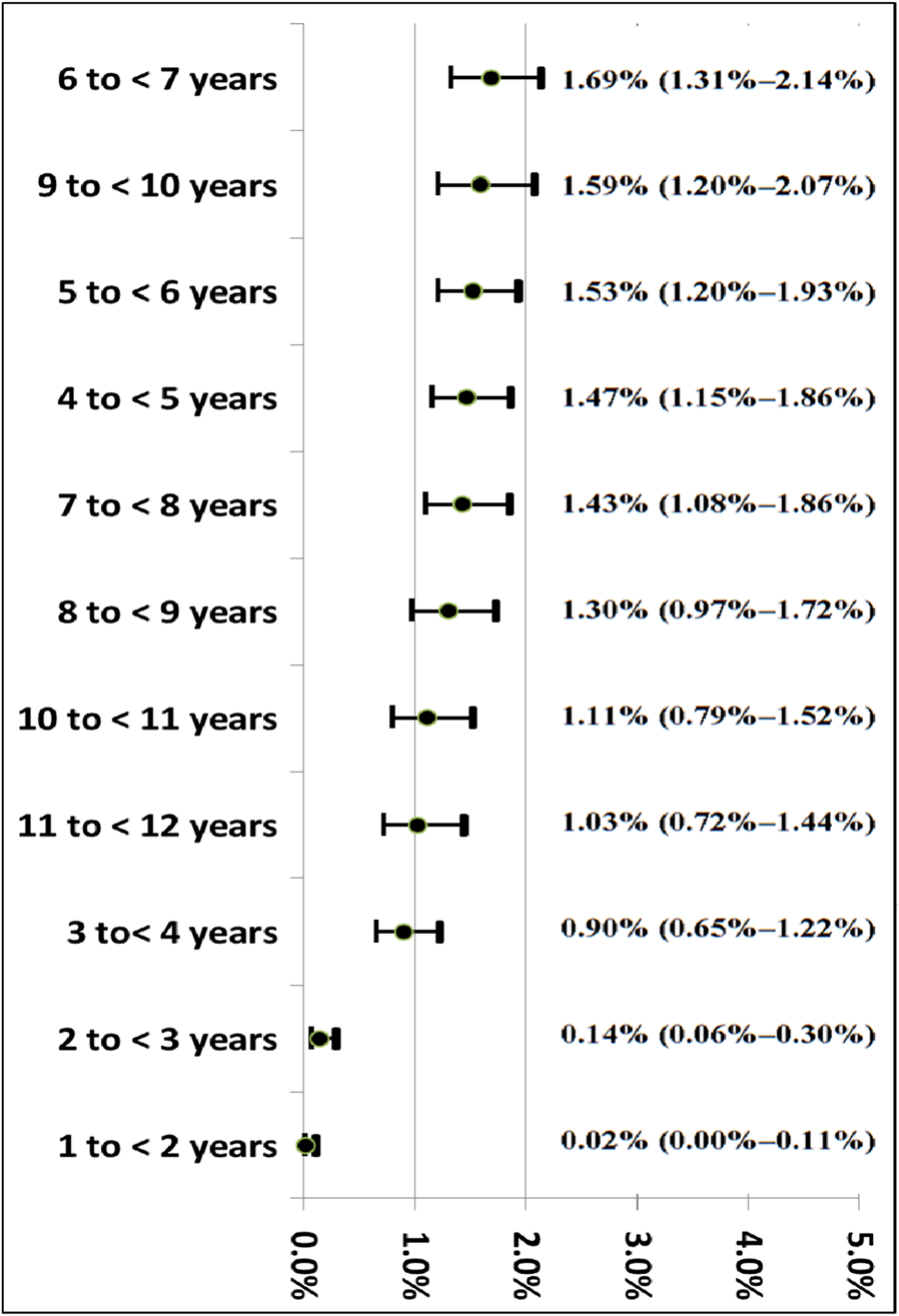
Prevalence plot of autism according to the highest prevalence/age, the overall prevalence among children aged 1–12 years (Combined sex) = 1.1% (95% CL: 1.0–1.2%), population survey

**Fig. 5:**
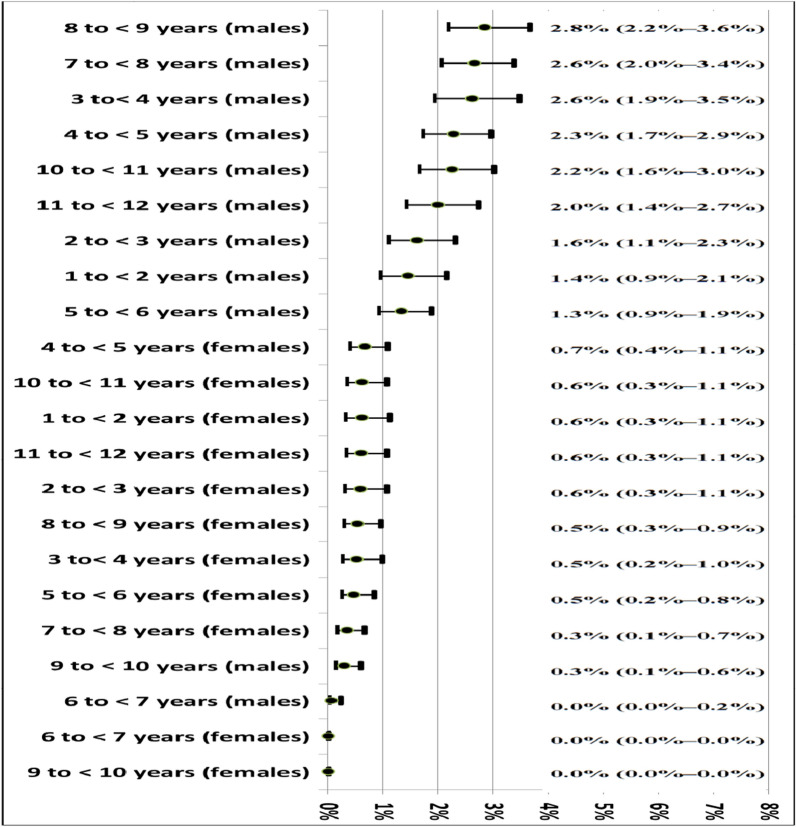
Prevalence plot of autism according to the highest prevalence/sex, the overall prevalence among male children aged 1–12 years = 1.7% (1.5%−1.9%), and among female children = 0.4% (0.3–0.5%), population survey

Figures 4 and 5 present age- and sex-specific estimates of autism prevalence. The highest prevalence was observed in the 6 to < 7-year age group (1.69%, 95% CI: 1.31%–2.14%), followed by the 9 to < 10-year age group (1.59%, 95% CI: 1.20%–1.93%). The lowest prevalence was recorded in children aged 1 to < 4 years, as shown in Fig. 4. The overall autism prevalence among children aged 1–12 years (both sexes combined) was 1.1% (95% CI: 1.0%–1.2%), based on the population survey.

Autism prevalence among boys was four times higher than among girls. The overall prevalence among males aged 1–12 years was 1.7% (95% CI: 1.5%–1.9%), while among females, it was 0.4% (95% CI: 0.3%–0.5%). The highest prevalence was observed in males aged 8 to < 9 years (2.8%, 95% CI: 1.32%–3.6%), followed by those aged 7 to < 8 years (2.6%, 95% CI: 2.0%–3.4%) and 3 to < 4 years (2.6%, 95% CI: 1.9%–3.5%). The lowest prevalence among males was in the 9 to < 10-year age group (0.3%, 95% CI: 0.1%–0.6%). Notably, no females with autism were identified in the 6 to < 7-year and 9 to < 10-year age groups, as shown in Fig. 5

In the multivariate logistic regression analysis presented in Table 3, the presence of ASD was associated with seven risk factors and two protective factors. The identified risk factors included the following:

- Convulsions After Birth: Children who experienced convulsions after birth had more than four times the odds of developing ASD compared to those without convulsions (AOR = 4.74; 95% CI: 3.3–6.8).
- Low Birth Weight: Babies weighing less than 2.5 kg at birth had increased odds of ASD (AOR = 2.08; 95% CI: 1.54–2.79).
- Family History of OCD: A family history of obsessive–compulsive disorder (OCD) was associated with twice the odds of having children with ASD (AOR = 2.07; 95% CI: 1.58–2.64).
- Prolonged Incubator Stay: Children who stayed in an incubator for more than two days had almost double the odds of developing ASD compared to full-term, healthy children (AOR = 1.91; 95% CI: 1.46–2.49).
- Maternal Health Problems: Mothers with any health problems during pregnancy had 1.5 times higher odds of having a child with ASD compared to healthy mothers (AOR = 1.66; 95% CI: 1.26–2.19).
- Male Gender: Males had 1.5 times greater odds of ASD than females (AOR = 1.62; 95% CI: 1.36–1.95).
- Urban Residency: Children in urban areas had 1.5 times the odds of developing ASD compared to those in rural areas (AOR = 1.5; 95% CI: 1.09–2.06).

**Table 3:** Multivariate Logistic regression model for predictors of ASD

Parameters	Diagnosed + ve ASD Vs. Screen -ve ASD		
	Wald	AOR	CI
*Sociodemographic factors*			
Sex (The base is: male)	28.799	1.69*	1.36–1.95*
Locality (urban – rural) (The base is: rural)	16.637	0.65	0.53–0.80*
demography (cities-lower Egypt-upper Egypt-frontiers) Cities to Frontiers (The base is: cities)	6.250	1.5*	1.09–2.06*
Paternal Education (The base is: university and above)	15.709	0.46*	0.31–0.68*
*Family, maternal and neonatal risk factors*			
Family history of obsessive–compulsive disorder (OCD)	10.95	2.07*	1.58–2.64 *
Mothers have any health problem during pregnancy	12.748	1.66*	1.26–2.19*
baby’s weight less than 2.5 kg at birth	23.351	2.08**	1.54–2.79*
Child suffer from convulsions after birth	71.342	4.74**	3.30–6.80*
Child kept in an incubator for more than two days	22.738	1.91*	1.46–2.49*

Model includes significant risk factors regarding children with or without ASD

Risk factors were entered into the multivariate logistic regression using (backward) selection procedure

^*^ = sig (< 0.05)

^**^ = highly sig (< 0.01)

### Protective factors

- Rural Residency: Living in a rural area decreased the odds of ASD by 30% compared to urban residency (AOR = 0.65; 95% CI: 0.53–0.80).
- Paternal Education: Having a father with a university degree or higher reduced the odds of ASD by 60% compared to those with lower education levels (AOR = 0.46; 95% CI: 0.31–0.68).

The severity of autism was assessed using both the DSM-5 and CARS, as shown in Table 4. According to the DSM-5 criteria, most participants were classified as having moderate ASD (45%), followed by severe ASD (39%) and mild ASD (16%). The severity distribution was similar between boys and girls, with no significant gender differences (*p* > 0.05). However, girls exhibited a slightly higher percentage of severe ASD compared to boys (42.5% vs. 38.3%) and marginally higher mean CARS scores. No significant differences were detected in severity levels across age groups.

**Table 4:** Distribution of severity of ASD per sex and age as diagnosed by DSM-5 and CARS

Variables totals (n = 455)	Levels of Severity			Test of sig. and (P value)
	Mild (n = 73) 16%	Moderate (n = 204) 45%	Severe (n = 178) (39%)	
Sex*				
Boys (n = 368) Girls (n = 87)	56(15.2%) 17(19.5%)	171(46.5%) 33(37.9%)	141(38.3%) 37(42.5%)	χ^2^ = 2.28 (P = 0.32)
CARS (Mean ± SD) Boys (n = 368) Girls (n = 87) Totals (n = 455)	37.76 ± 5.4 39.69 ± 7.18 37.94 ± 5.77			t = 1.33 (P = 0.181)
*Age group*				
1–3 years (n = 45) > 3–6 years (n = 194) > 6–12 years (n = 216)	11(24.4%) 28(14.4%) 34(15.7%)	18 (40%) 90(46.4%) 96(44.4%)	16(35.6%) 76(39.2%) 86(39.8%)	χ^2^ = 2.83 (P = 0.587)
CARS (Mean ± SD) 1–3 years (n = 45) > 3–6 years (n = 194) > 6–12 years (n = 216)	37.41 ± 4.9 37.58 ± 5.2 38.38 ± 6.5			F = 1.166 (P = 0.312)

+ test of significance between groups

A total of thirty-three significant variables associated with autism severity were analyzed using multivariate logistic regression with a backward selection procedure, as presented in Table 5. These variables were categorized into risk factors for autism severity, encompassing seven sociodemographic/family factors, eleven maternal risk factors during pregnancy, four obstetric/perinatal variables, three family history factors, and eight environmental risk variables.

**Table 5:** Multivariate Logistic regression model for predictors of severity of autism spectrum disorder (Severe vs. non-severe)

	Wald	*P*-value	AOR	CI
Governorate				
Fayoum	10.58	0.001*	6.6	2.12–20.79
Dakahlia	4.08	0.043*	0.384	0.15–0.97
Damietta	6.09	0.014*	0.32	0.13–0.80
Age (get older)	13.81	< 0.001**	1.17	1.08–1.27
Sex (female)	29.38	< 0.001**	3.33	2.15–5.14
Influenza during pregnancy	18.12	< 0.001**	2.191	1.09–2.41
Maternal hypertension	10.56	0.001**	1.174	1.06–1.5
Mothers taking anti-emetic drugs	10.39	0.001**	1.48	1.31–1.75
Mothers taking antibiotic drugs	14.76	0.029*	1.58	1.36–1.95
Mothers taking hypoglycemic drugs	16.72	0.01**	2.07	1.01–2.52
Children with history of cyanosis after birth	10.12	0.001**	1.46	1.28–1.74
Neonatal jaundice that necessitated hospitalization	21.13	< 0.001**	2.35	1.22–2.54
Hospitalization of the child being kept in an incubator for more than two days after birth	35.03	< 0.001**	4.69	2.81–7.83
Family history of epilepsy	15.59	< 0.001**	2.15	2.06–2.38
Family history of obsessive–compulsive disorder (OCD)	14.16	0.41*	3.16	1.05–9.57
Family history of depression	12.85	< 0.001*	2.06	2.14–2.29
Obtaining water from tap outside the house	6.89	0.009**	7.5	1.67–33.75
Painting condition of the house is not good	6.69	0.01*	9	1.7–47.6

Model includes significant risk factors regarding severe versus non-severe, * = sig (< 0.05), ** = highly sig (< 0.01)

The final model demonstrated a good fit and identified 15 risk factors that were associated with more than twice the odds of severe autism. These factors, listed in order of their odds ratios, included:

- Painting Condition of the House: Poor condition (AOR = 9; 95% CI: 1.7–47.6)
- Water Source: Obtaining water from a tap outside the house (AOR = 7.5; 95% CI: 1.67–33.75)
- Governorate: Living in Fayoum (AOR = 6.6; 95% CI: 2.12–20.79)
- Hospitalization: Incubator stay for more than two days after birth (AOR = 4.69; 95% CI: 2.81–7.83)
- Gender: Being female (AOR = 3.33; 95% CI: 2.15–5.14)
- Family History of OCD: (AOR = 3.16; 95% CI: 1.05–9.57)
- Neonatal Jaundice: That necessitated hospitalization (AOR = 2.35; 95% CI: 1.22–2.54)
- Family History of Epilepsy: (AOR = 2.15; 95% CI: 2.06–2.38)
- Family History of Depression: (AOR = 2.06; 95% CI: 2.14–2.29)
- Maternal Influenza: During pregnancy (AOR = 2.19; 95% CI: 1.09–2.41)

Factors associated with approximately 1.5 times the odds of severe autism included a history of cyanosis after birth and the use of antibiotic or antiemetic drugs. Additionally, age and maternal hypertension significantly increased the risk of severe autism. However, children residing in Lower Egypt (Dakahlia or Damietta) were less likely to exhibit severe autism.

## Discussion

ASD is a highly disabling developmental disorder, imposing a significant economic burden. Accurate prevalence estimates are essential for governments to formulate effective intervention policies for individuals with ASD and their families [70]. This study is the first nationwide analysis of ASD prevalence in Egypt, covering all governorates and not limited to specific groups of patients. It estimates the national prevalence of ASD in children aged 1–12 years using DSM-5 criteria and CARS while exploring geographical disparities in ASD prevalence.

### Prevalence of ASD in Egypt and comparative analysis

This study evaluated 41,640 children, estimating ASD prevalence at 1.1% (455 cases). Statistically significant differences in ASD prevalence were observed between Egyptian governorates. ASD prevalence varies significantly across world regions [71]. Studies indicate higher ASD rates in developed countries compared to resource-limited regions [72]. The global ASD prevalence ranges from 1.09 to 436.0 per 10,000 children, with a median prevalence of 100 per 10,000 [73].

Qatar reported 1.14% ASD prevalence among school children aged 6–11‐years [74], while Oman [75] reported 20.35 per 10,000 among children aged 0–14 years referred to autism diagnostic centers. Saudi Arabia [76] reported 2.81 per 1,000 among children aged 6–12 years in Makkah and Jeddah. The prevalence found in this study aligns with rates reported in Europe and Qatar [74, 77]. Previous Egyptian studies did not establish a clear ASD prevalence due to variations in study design and context. A facility-based study in one Egyptian governorate (500 children, ages 3–12) reported an ASD prevalence of 3.4% [24].

### Specific diagnostic tools and screening methods influencing prevalence rates

Diagnosing ASD is a challenging process due to the absence of specific biological markers. Owing to the complexity of the ASD pathophysiology, the diagnostic process remains predominantly descriptive rather than explanatory. It usually depends on information from two primary sources: parent or other caregiver descriptions of the child’s development and meticulous observations of the child’s behavior by an expert clinician [78]. Varying sensitivities and specificities among tools for diagnosing ASD leads to screening errors and imprecise diagnosis. This difficulty emphasizes the necessity of extremely sensitive and age-appropriate techniques for efficient ASD screening and diagnosis.

The M-CHAT is the most widely used screening tool with a sensitivity and specificity ranged from 87%−91% (53). Standardized criteria for diagnosing ASD are provided by the Diagnostic and Statistical Manual, Fifth Edition (DSM-5) published by the American Psychiatric Association [1]. Among other sensitive diagnostic tools, ADOS showed a sensitivity of 87% (95% CI 0.79‒0.92) and specificity 75% (95% CI 0.73‒0.78); ADI-R demonstrated test sensitivity of 77% (95% CI 0.56‒0.90) and specificity 68% (95% CI 0.52‒0.81), CARS test sensitivity was 89% (95% CI 0.78‒0.95) and specificity 79% (95% CI 0.65‒0.88) [79]. A consistent prevalence was obtained in the current national survey by selecting a range of valid and reliable screening and diagnostic tools that were age-appropriate.

### Gender disparities in ASD prevalence

The study revealed that the prevalence of ASD was four times higher in boys (1.7%) than in girls (0.4%). Further analysis showed that boys had 1.69 times greater odds of being diagnosed with ASD compared to girls (95% CI: 1.36–1.95). These findings align with global research, where the median boy-to-girl ratio in ASD prevalence is approximately 4.2:1. In the USA, ASD is reported to be 3.8 times more common among boys than girls, while the UK shows a similar ratio of 4:1 [80].A meta-analysis of 54 studies (13,784,284 participants) estimated the true male-to-female ratio closer to 3:1 [81]. Being a female was considered a protective factor [82, 83]. Developmental delays in speech, language, and communication skills are also more commonly observed in boys [35], potentially due to elevated fetal testosterone levels [84].

### Age-specific diagnostic challenges and trends

Two core criteria must be met for an ASD diagnosis: persistent impairments in social communication and restrictive, repetitive behaviors. Symptoms present differently across age groups: In infancy, the distinguished behaviors may be lack of following objects visually, avoidance to hugging or the child may not point to objects. In preschoolers, the presentation may be poor eye contact, speech delay, high sensitivity to sounds or bright light, while in school-age children, the notable features may be delays in language processing, rigid behaviors and resistance to group activities. In the current study, the researchers have used standardized screening and diagnostic tests appropriate to each age category.

### Age-related trends in ASD prevalence

The study used age-appropriate standardized screening and diagnostic tools. ASD prevalence was highest in children aged 3–6 years (1.50%), consistent with findings from other studies [30]. Recent literatures have reported the possibility of ASD diagnosis in the early years of life, even in the first six months with a good prognosis [85]. Factors contributing to this peak include lack of stimulating environments [86, 87], malnutrition with increased vulnerability to diseases [88, 89] due to defective weaning procedures or the unhealthy dietary behavior [90]. Parents often don’t recognize ASD signs in infancy, as delayed speech and social interaction become more evident in preschool years. Conversely, mild ASD symptoms may improve in school-age children, leading to slightly reduced prevalence. In the Arab world, limited child mental healthcare often results in delayed ASD diagnosis and treatment [91].

### Geographic disparities in ASD prevalence

This study found higher ASD prevalence in urban areas than in rural regions. Cairo had the highest confirmed ASD prevalence at 2.5%. Similar patterns have been observed in the USA, France, Sweden, and Japan, where ASD rates in urban areas were 2.5 times higher than in rural areas [92]. Studies from China and Denmark reported similar findings [93, 94]. Potential contributing factors include: urban stress exposure with maternal stress and immune dysregulation during pregnancy may be significant ASD risk factors [95]. Air pollution which affects neurodevelopment via epigenetic modifications, neuroinflammation, oxidative stress, and endocrine disruption [96]. On the other hand, a Greek study found no urban–rural ASD prevalence difference [97], this may be due to exclusion of major cities from their analysis.

Due to several interrelated variables [98–100], most children with ASD living outside cities receive diagnosis later in life. The healthcare system required to assess, diagnose, and support their medical requirements is not usually available. Even physicians may not possess specific knowledge required to screen, diagnose, and refer individuals with ASD. Parents of children with ASD may be discouraged from seeking medical advice outside their community and interacting with the healthcare system due to high costs, long distances, and lack of awareness of ASD signs. Stigma is an additional obstacle [101]

### Socioeconomic factors and parental education in ASD prevalence

This study found that higher parental education (University graduated or above) was associated with a 60% decrease in the odds of having a child with ASD (AOR = 0.46, 95%, CI 0.31–0.68). Parental education is one of the environmental factors associated with ASD. Other significant environmental influences may include prenatal, perinatal, and postnatal conditions. Highly educated parents can take proactive measures to reduce the risk of autism by prioritize genetic counseling and testing, focusing on prenatal care and nutrition, and avoiding environmental toxins. These strategies may help reduce the risk of autism [102]. These findings are consistent with other studies that have identified parental illiteracy as a strong risk factor for ASD [103–105]. Otherwise, highly educated parents may recognize ASD signs earlier, which could contribute to higher ASD prevalence in this group [106]. However, ASD is still a complex condition with a multifactorial etiology.

### Egypt-specific factors influencing prevalence rates

Family, maternal, and neonatal risk variables were identified as the main predictors of ASD in Egypt by the multivariate logistic regression analysis.

#### Maternal and neonatal risk factors

Multivariate regression identified maternal and neonatal health as key ASD predictors. Maternal health issues during pregnancy significantly increased ASD odds (AOR = 1.66; 95% CI 1.26–2.19), aligning with prior research [107]. A Chinese study found maternal stress doubled ASD risk, potentially due to disrupting prenatal testosterone exposure, which plays a neuroprotective role [25].

Neonatal complications, including prematurity, low birth weight, and jaundice, were also associated with increased ASD risk, consistent with previous findings [108, 109]. These results emphasize the importance of maternal and neonatal health in ASD prevention [23]. Egypt’s community-based health interventions have improved maternal and neonatal outcomes, but further efforts are needed [110, 111].

Family history of OCD, schizophrenia, and depression was linked to increased ASD risk and severity. Similar associations have been reported in previous studies [112]. Maternal fever, infection, and medication use during pregnancy also elevated ASD risk, as observed in other studies [113]. Janecka et al., (2018) suggested that maternal health status, rather than medication use, is a key ASD risk factor [114]. Maternal infections may alter cytokine levels, affecting fetal neurodevelopment [115].

#### ASD severity and environmental factors

The severity of autism in the children studied was assessed using both DSM- 5 and CARS. According to DSM-5, nearly half of the children (45%) had moderate ASD, 39% had severe ASD, and 16% had mild ASD. The distribution of ASD severity was similar across age groups and gender with no significant statistical difference (*p* > 0.05). On the contrary, an Italian study revealed that 21–16% of children with autism had mild to moderate ASD, while 63% had severe ASD [116]. Similarly, El Baz and his colleagues [117] found that 43% of participants had mild to moderate ASD, and 57% had severe ASD. Assessing ASD severity in this study serves as a crucial step for parents to initiate effective training programs [118]. Risk factors for ASD severity were identified through multivariate regression analysis.

Environmental factors have emerged as strong predictors of ASD severity in this study. Poor house painting conditions (AOR = 9; 95% CI 1.7–47.6) and obtaining water from a tap outside the house (AOR = 7.5; 95% CI 1.67–33.75) were the most prominent predictors. However, the wide confidence intervals suggest that these estimates should be interpreted with caution. While the lower bound suggests a moderate effect, the upper bound implies an extremely strong association. This variability highlights the need for larger sample sizes, more detailed environmental exposure assessments, and further replication studies to refine the precision of these estimates. Future research should consider stratified analyses or Bayesian modeling approaches to mitigate uncertainty and improve effect size estimation.

There is evidence that exposure to chemical pollutants at critical developmental stages may affect neural and behavioral development [119]. Environmental factors such as poor housing conditions and contaminated water sources may exacerbate ASD severity through multiple biological mechanisms. Neurotoxicant exposure is one of the primary pathways, where heavy metals such as lead, arsenic, and mercury, commonly found in deteriorating housing materials and unsafe water supplies, impair neuronal signaling and synaptic plasticity. These toxicants also induce oxidative stress, leading to neuronal damage and increased ASD symptom severity [120, 121]. Oxidative stress and mitochondrial dysfunction further contribute to ASD progression, as pollutants and pesticides trigger excessive production of reactive oxygen species (ROS), which disrupts neuronal energy metabolism and leads to cognitive and behavioral impairments [122, 123]. Another significant mechanism is immune dysregulation and neuroinflammation, where environmental toxins activate pro-inflammatory cytokines such as IL-6 and TNF-α, which in turn lead to chronic neuroinflammation, worsening ASD-related behavioral and cognitive deficits [124, 125]. Epigenetic modifications also play a crucial role, as exposure to heavy metals and environmental pollutants can alter DNA methylation and histone modifications, affecting the expression of genes critical to neurodevelopment, thus influencing ASD severity [126, 127]. Additionally, gut microbiome disruptions have been linked to ASD, as contaminated water sources can introduce pathogenic bacteria and toxins that alter gut microbiota composition, increase gut permeability, and promote systemic inflammation, all of which negatively impact brain function and ASD symptoms [128, 129].

### Policy implications and recommendations

#### Strengthening early detection and diagnosis

Early detection of ASD is crucial for timely intervention and better developmental outcomes. This study highlights the need for improved screening programs, particularly for high-risk groups, including children with neonatal complications and those from socioeconomically disadvantaged backgrounds. Integrating ASD screening into routine maternal and child health services can facilitate early identification, ensuring children receive appropriate interventions at a critical developmental stage.

Additionally, training healthcare professionals, including pediatricians and primary healthcare workers, is essential for improving early referral systems and reducing diagnostic delays. Expanding access to screening tools in primary healthcare facilities will ensure that children with ASD, especially in underserved areas, are not overlooked.

#### Enhancing maternal and neonatal healthcare

Findings from this study demonstrate a strong association between maternal health conditions, perinatal complications, and the increased risk of ASD. Maternal infections, difficult labor, and neonatal distress have been linked to neurodevelopmental disorders, necessitating improvements in prenatal and neonatal healthcare. Strengthening maternal healthcare services, particularly in rural and low-income areas, can help mitigate these risks.

Public health campaigns should focus on educating expectant mothers about ASD-related risk factors, promoting early interventions during pregnancy, and ensuring that neonates with perinatal complications receive appropriate postnatal care. Expanding neonatal intensive care unit (NICU) services and implementing standardized follow-up protocols for high-risk infants will further support early developmental monitoring and intervention.

#### Expanding educational and social support systems

Children with ASD, particularly those from low-income and low-education households, face significant barriers in accessing specialized education and therapy. Inclusive education policies must be strengthened to integrate children with ASD into mainstream schools, ensuring that teachers receive adequate training to support neurodiverse students. Financial support for therapy can improve developmental outcomes.

Establishing national ASD resource centers can provide caregivers with essential support services, including financial aid, respite care, and therapy options. Continuous research and data collection on ASD prevalence and intervention outcomes should be prioritized, with a national autism registry facilitating long-term tracking of prevalence rates, risk factors, and intervention effectiveness.

### Bridging urban–rural disparities

Targeted training programs for pediatricians, general practitioners, and community health workers should be implemented nationwide, with an emphasis on early detection protocols and ASD management. Regional healthcare centers should be equipped with standardized diagnostic tools, such as M-CHAT and CARS, to ensure that all healthcare professionals have access to validated screening measures. A structured referral system should also be established between primary healthcare units (PHUs) and tertiary centers specializing in neurodevelopmental disorders to facilitate timely diagnosis and intervention.

Given the geographical disparities in healthcare access, telemedicine and digital health platforms could be leveraged to provide remote consultations, parent training, and specialist referrals for ASD management. Virtual ASD assessment programs can help rural primary care centers connect with specialists in tertiary hospitals, reducing the burden of travel and wait times for families. Additionally, mobile health (mHealth) applications that provide caregivers with educational resources, digital screening tools, and access to telehealth consultations can improve early detection and intervention outcomes in remote regions.

### Ensuring regional equity in ASD intervention

Access to ASD-specific therapies, including behavioral therapy, speech therapy, and occupational therapy, remains largely concentrated in urban centers, leaving children in rural areas with limited intervention options. Decentralizing ASD intervention services by integrating them into regional rehabilitation centers, community health clinics, and educational institutions will ensure that children with ASD in all regions receive appropriate support. Additionally, financial incentives, such as government subsidies and health insurance coverage, should be expanded to lower-income families to ensure that ASD treatment is not restricted to those who can afford private care. Meanwhile, a national autism registry with region-specific data on prevalence and interventions can guide resource allocation and policy decisions, ensuring equitable ASD care across Egypt.

### Addressing environmental and socioeconomic disparities

This study identifies environmental and socioeconomic factors as key determinants of ASD prevalence and severity. Poor housing conditions, exposure to environmental toxins, and limited access to clean water have been associated with a higher likelihood of severe ASD symptoms. Improving housing regulations and ensuring access to safer living environments can help mitigate these risks. Additionally, parental education plays a significant role in reducing ASD prevalence, as higher education levels are linked to better prenatal care, enriched home environments, and early intervention. Expanding community-based parental education programs can provide families with the necessary knowledge and resources to support their child’s development. Reducing urban healthcare disparities by establishing specialized ASD care centers in underserved areas and leveraging telemedicine for remote consultations will enhance healthcare accessibility for families affected by ASD.

### Strengths

This study has several notable strengths. To our knowledge, it is the first study conducted on a national level in Egypt with a large, representative sample of Egyptian children aged 1–12 years. The sample represents all Egyptian governorates and various socioeconomic classes, making the findings generalizable to the entire country. Moreover, national intervention programs can be tailored based on the prevalence rates reported for each governorate.

The study’s accuracy is high, given the large sample size, small margin of error, and high confidence level. A wide range of epidemiological, sociodemographic, environmental, and medical risk factors were identified, offering insights for primary prevention measures. Additionally, the study highlights the most vulnerable children, who may benefit from further monitoring for potential ASD diagnoses. Lastly, the assessment of ASD severity provides a valuable foundation for parents and healthcare providers to implement precise intervention programs.

#### Limitations

This study has certain limitations. Due to the large sample size, it was not feasible to perform comprehensive assessments for the entire population. However, this limitation was mitigated by conducting the study in four phases, culminating in confirmed diagnoses through standardized tests.

Although environmental risk factors, such as metal toxicity, were identified, their verification through laboratory investigations was beyond the study’s budget. Unfortunately, the Autism Diagnostic Observation Schedule, 2nd (ADOS-2)—the “gold standard” for ASD diagnosis—could not be included due to its high cost [130] and the time required for administration (40–60 min by experienced evaluators) [131]. Although more expensive than the Childhood Autism Rating Scale (CARS), ADOS-2 offers similar levels of sensitivity and specificity, as confirmed by a recent systematic review, which recommended the use of either test for ASD diagnosis [79].

Additionally, the cross-sectional design of the study restricts its ability to track trends in autism spectrum disorder (ASD) over time. While this design allows for the calculation of prevalence and the measurement of autism’s burden, it restricts the ability to make a causal inference. Additionally, risk assessment may be subject to nonresponse bias and recall bias.

To mitigate recall bias, the research team sought corroborative information from multiple family members (e.g., father or grandmother) or from alternative sources (e.g., medical reports) whenever possible. Moreover, the precise three-stage sampling procedure helped reduce surveillance bias, ensuring a robust and representative dataset.

## Conclusion

The national prevalence of ASD in Egypt had not been previously reported, making this study the first of its kind. This cross-sectional national survey used a representative sample from eight governorates, covering Egypt’s major geographic regions. By identifying risk factors, this study offers new opportunities to explore the gene-environment interplay that may contribute to ASD development. The assessment of ASD severity is particularly significant. Early detection, diagnosis, and intervention for infants are crucial to reducing both individual suffering and social costs.

### Recommendations

The findings of this study will help guide efforts toward preventive measures and early interventions to reduce the incidence of ASD and other neurodevelopmental disorders. National intervention programs should be tailored to the specific prevalence reported in each governorate. Primary prevention efforts should target all families by ensuring access to quality antenatal care for pregnant women, particularly in rural areas, Upper Egypt, and frontier governorates. Additionally, ASD screening and surveillance should be implemented in maternal and child health care centers across the country. Health professionals must be trained to recognize early signs of ASD, and preschool children (aged 3–6) should receive focused attention through early childhood development programs in nurseries and child care centers. Parenting education programs should also be expanded to promote positive parenting practices.

Secondary prevention should focus on families with identified risk factors, such as premature or low birth weight infants, or those with neonatal complications like cyanosis or convulsions. Interventions should also address modifiable environmental risk factors to mitigate ASD risk.

For children already diagnosed with ASD, tertiary prevention measures should include providing free or low-cost services such as speech therapy, physiotherapy, occupational therapy, and sensory integration sessions, ensuring comprehensive support for both children and their caregivers.

## Research recommendations for future studies

To build on this study’s findings, future research should prioritize longitudinal studies to establish causal relationships between environmental exposures, perinatal factors, and ASD severity. Investigating genetic and epigenetic influences will provide insights into gene-environment interactions in ASD risk, particularly in the Egyptian population. Additionally, further research should explore the impact of environmental pollutants, such as heavy metals and industrial toxins, on neurodevelopmental outcomes to guide risk mitigation strategies.

Studies addressing regional and socioeconomic disparities in ASD diagnosis and healthcare access are also crucial. Understanding barriers to early detection and intervention in rural versus urban areas can help policymakers improve healthcare equity. Research evaluating the effectiveness of early intervention programs, such as behavioral therapy and parental training, should be conducted to optimize treatment approaches and improve developmental outcomes.

Emerging areas of interest, such as the role of gut microbiome alterations and nutritional factors in ASD severity, require further investigation. Analyzing gut microbiota profiles in ASD-affected children and assessing dietary interventions may provide novel therapeutic strategies. Additionally, establishing a national autism registry will enhance data standardization, track prevalence trends, and support policy-driven healthcare improvements.

Lastly, exploring gender-based differences in ASD presentation and diagnosis is essential to ensure that diagnostic tools and intervention strategies are inclusive of both boys and girls. Addressing these research gaps through collaborative studies, standardized data collection, and policy-driven research initiatives will significantly enhance ASD early detection, risk assessment, and intervention effectiveness in Egypt and similar regions.

## Supplementary Information

Supplementary material 1.

## Data Availability

All data generated or analyzed during this study are available upon reasonable request.

## Abbreviations

ASD: Autism Spectrum Disorder
DSM-5: Diagnostic and Statistical Manual of Mental Disorders, Fifth Edition
CARS: Childhood Autism Rating Scale
CAPMS: Central Agency for Public Mobilization and Statistics
VABS: Vineland Adaptive Behavior Scales
VABSA: Arabic Version of Vineland Adaptive Behavior Scales
M-CHAT: Modified checklist for Autism in Toddlers
GARS-2: Gilliam Autism Rating Scale
MOH: Ministry of Health
CDC: Cairo Demographic Centre
DHS: Demographic and Health Survey in Egypt
NRC: National Research Center of Egypt
MOHP: Ministry of Health and Population
MCH: Maternal and Child Health
