# Supplementary material for: Mapping autism in Egypt: population-based insights into prevalence, risk determinants, and severity among children aged 1–12 years
Source: Mol Autism. 2025 May 29;16:32. doi: 10.1186/s13229-025-00665-1 (PMC12121136; doi:10.1186/s13229-025-00665-1)
Supplement: Supplementary file 1 — Supplementary material 1. [file 13229_2025_665_MOESM1_ESM.doc]

**S-Table-1: List of the targeted Households (HH) according to the governorates, locality and sociodemographic status for screening of autism among children aged 1-12 years**

| serial | **Gov.** | **classification according to HDI** | **Kesm/Markaz** | **Urban** | | **Rural** | | | **Total** |
| --- | --- | --- | --- | --- | --- | --- | --- | --- | --- |
|  |  |  |  | **Shiakha (English)** | **HH** | **Local Unit ( (English )** | **Village (English )** | **HH** | **HH** |
| **1** | **Cairo** | **High** | **AlNozhah** | **Al Hicksit** | **1390** |  |  |  | 1390 |
|  |  | **middle** | **AlSaiedah Zainab** | **Alkabsh** | **1390** |  |  |  | 1390 |
|  |  | **low** | **AlSharabia** | **Al Amiria** | **1390** |  |  |  | 1390 |
| **2** | **Dakhlya** | **High** | **AlSenbelawin** | **Al Sinblaween city** | **317** | **Kafr Alruwk** | **Alshalaa** | **905** | 1222 |
|  |  | **middle** | **MietSalsil** | **Mit salsil city** | **317** | **Alatihad** | **AlJafara** | **905** | 1222 |
|  |  | **low** | **AlMataria** | **Almataria city** | **317** | **Alsafra** | **Al Dahear** | **905** | 1222 |
| **3** | **Gharbia** | **High** | **KafrElZaiat** | **KafrElZaiat** | **260** | **Kafour Belshay** | **Qasta** | **680** | 940 |
|  |  | **middle** | **Samanood** | **Samanood** | **260** | **Ziyad’s locality** | **Munshat Nzif** | **680** | 940 |
|  |  | **Low** | **Markaz of Qutour** | **Qutour** | **260** | **Kotour** | **Khabata** | **680** | 940 |
| **4** | **Fayoum** | **High** | **Markaz of Al Fayoum** | **Alqism rabie** | **145** | **Dacia** | **Al Sunbat** | **580** | 725 |
|  |  | **middle** | **Markaz of**  **Senoures** | **Senoures** | **145** | **Terrsa** | **Alzawia El Khadra** | **580** | 725 |
|  |  | **Low** | **Markaz of Tamiaha** | **Tamiaha** | **145** | **Sarsna** | **Kafr Omira** | **580** | 725 |
| **5** | **Assuit** | **High** | **Hay Shark** | **Alwalidia Alwustania** | **235** | **Bani Hussein** | **Musriea** | **795** | 1030 |
|  |  | **middle** | **Al Kousiah** | **Al Kousiah City** | **235** | **Mir** | **Bani Hilal** | **795** | 1030 |
|  |  | **Low** | **Al Ghanaiem** | **Al Ghanaiem** | **235** | **Alazayiza** | **Al Amri** | **795** | 1030 |
| **6** | **Aswan** | **High** | **Nasr Al Nouba** | **Nasr Al Nouba City** | **280** | **Korta** | **Garf Hussein** | **410** | 690 |
|  |  | **middle** | **Edfo** | **Al-Busaliya Bahri** | **280** | **Alramad Albahry** | **Adfu Quabli** | **410** | 690 |
|  |  | **Low** | **Markaz KoomOmbo** | **KoomOmbo** | **280** | **Al Abbasia** | **Sabaa Quabli** | **410** | 690 |
| **7** | **Damietta** | **High** | **AlRawda** | **AlRawda** | **275** | **Hajaja Village** | **Hajaja Village** | **380** | 655 |
|  |  | **middle** | **Al zarqaa** | **Alsarw** | **275** | **Sharmsah** | **Kafr Toqaa** | **380** | 655 |
|  |  | **Low** | **Kafr-Saad** | **Kafer Albatiykh** | **275** | **Kafr Saad Country** | **Nawasiriya village** | **380** | 655 |
| **8** | **MarsaMatrouh** | **High** | **Marsa Matrouh** | **MarsaMatrouh**  **(Alsanusia & Kilo 4)** | **470** | **Alkasr** | **Alkasr** | **220** | 690 |
|  |  | **middle** | **Al Hamam** | **Al Hamam City** | **470** | **Alsalam** | **Alsalam** | **220** | 690 |
|  |  | **Low** | **AlNajyla** | **AlNajyla** | **470** | **Almathany** | **Almathany** | **220** | 690 |
| **Total** | |  | | | **10116** |  | | **11910** | **22026** |
